# Single and combined effects of *CSN1S1* and *CSN2*-casein genes on Awassi sheep milk quantity and quality

**DOI:** 10.14202/vetworld.2022.435-441

**Published:** 2022-02-25

**Authors:** Ahmad H. Al-Amareen, Khaleel I. Jawasreh

**Affiliations:** 1 Livestock Directorate, National Agriculture Research Center, Albaqa’a 19381, Jordan; 2 Department of Animal Production, Jordan University of Science and Technology, Irbid 22110, Jordan

**Keywords:** Awassi sheep, fat, gene, interaction, milk yield, protein

## Abstract

**Background and Aim:**

Milk produced from Awassi sheep is of high nutritive value; its production is relatively low in Awassi sheep, so the genetic improvement programs targeted milk production and its components are of high importance, especially when using genes that have an important signal to milk traits. This study was aimed at assessing the influence of alpha S1 (*CSN1S1*) and beta-casein (*CSN2*) genes genotypes interaction on Awassi ewes milk productivity.

**Materials and Methods:**

A total number of 391 milk yield and its composition records (taken through five consecutive years, 2007-2011) of 167 ewes were utilized for this study. DNA samples were extracted from the ewe’s blood samples, then the polymerase chain reaction products of alpha S1 (*CSN1S1*) and beta-casein (*CSN2*) genes were sequenced. The obtained sequences were analyzed; thereafter, the detected variants were tested for their possible association with milk traits.

**Results:**

The *CSN1S1* and *CSN2* variants allelic frequencies were 0.85 and 0.15, and 0.95 and 0.05, respectively. Lactose and solid not fat (SNF) % were associated with TC *CSN1S1* genotypes. No association was found among *CSN1S1* polymorphic genotypes with milk production, lactose, and SNF % were associated with TC *CSN1S1* genotypes. Ewes of *CSN2* AC genotype showed higher milk production traits, while no association was found between milk composition traits and *CNS2* genotypes. Nevertheless, CSN1S1*CSN2 interaction showed the highest SNF, fat percentages, and milk production.

**Conclusion:**

The substantial interaction effects between *CSN1S1×CSN2* genes were significantly affected the amount of milk, fat, and SNF% produced. The detected variants should be included in the breeding programs of Awassi sheep that are designed for improving their milk quantity and quality.

## Introduction

The potential use and incorporation of the genes that encoded milk proteins in breeding programs are of high importance; their pivotal role in enhancing the productivity and the manufacturing properties of sheep’s milk is highly recommended [1-3]. The mammary glands found in the ewe’s udder secret milk proteins by its epithelial cells [4]. The most abundant proteins found in sheep’s milk is the caseins (80%) in addition to whey proteins. Four groups of caseins were found, namely, alpha-S1-(*CSN1S1*), alpha-S2-(*CSN1S2*), beta-(*CSN2*), and kappa-(*CSN3*) caseins [4,5]. The genetic code of the casein proteins (250 kb) is located on chromosome number 6 of the ovine genome [6]. Moioli *et al*. [7] and Gras *et al*. [8] reported missense variants that cause changes in the protein’s amino acid which, directly altered the protein’s structure, some missense mutations, or variants have been observed to affect milk quality and quantity. Furthermore, the quality and the yield of cheese were improved following including casein variants into the selection programs of dairy sheep.

Due to its extraordinary micelle structure, Alpha S1-casein coded by the *CSN1S1* gene plays a pivotal role in yielding high amounts of cheese [5,9]. Chessa *et al*. [10] indicated *CSN1S1* protein to account for 47.21% of ovine whole milk proteins. At the DNA level, one missense variant (rs420959261) was detected within the *CSN1S1* gene in exon number 17. Ceriotti *et al*. [11] reported a mutation in this single nucleotide polymorphism (SNP) that causes substitution in p. Thr186Ile which intern change the CSN1S1*T protein variants from T to C. Ruminants milk components such as milk fat and protein contents in addition to cheese yield were observed to be highly associated with different *CSN1S1* variants as stated by Giambra *et al*. [12] and Moioli *et al*. [7]. Beta-casein constitutes about 50% of the total proteins found in milk, which, encoded by *CSN2* gene that has a pivotal role in the manufacturing properties of milk such as the formation process of micelles and its stabilization [5]. Moreover, *CSN2* is a member of casein cluster of 13 known protein variants; it is the most polymorphic milk protein gene. Due to its unique specifications, *CSN2* may act as a powerful biological marker for improving milk traits. The complete nucleotide sequence of *CSN2* was described by Provot *et al*. [13]. As appeared in the NCBI, the *CSN2* gene contains 9 exons; the largest exons are 7 and 9 and 492 and 323 bp in size, respectively. *CSN2* gene contains an SNP (rs430298704) which, substitute p. Met183Val that alter A and G protein alleles in addition to another one called rs416941267) [14]. Chessa *et al*. [10] reported a missense mutation (rs416941267) in the *CSN2* that changed C to A protein alleles in*CSN2*. The molecular characterization and the association between *CSN2* polymorphisms with milk performance were studied in sheep [12,15,16].

This is the first study designed to screen the commercial Awassi sheep *CSN1S1* and *CSN2* genes loci and investigate their single and combined effects on CSN2 genes and their association with milk production and composition.

## Materials and Methods

### Ethical approval

The procedures used in this project were approved (approval No. 16/3/3/578) by the Animal Care and Use Committee of Jordan University of Science and Technology.

### Study period and location

The study used the records of 2007-2011. The study was conducted in the commercial sheep flocks distributed in the southern, middle and Northern regions of Jordan.

### Animal and performance data

The full details of the fieldwork description, milk quantities and qualities, samples collection, analysis, and the studied flocks’ management were described in detail by Jawasreh *et al*. [17]. Out of 928 ewes available for this project, 391 full lactation records on 167 ewes were selected according to their measurement accuracy and the availability of pedigree information. All the inclusion and exclusion criteria are described in the study of Jawaresh *et al*. [17].

### DNA isolation and DNA segment amplification

Blood samples (5 mL) from each of 167 Awassi ewes were drained from the jugular vein using vacuum tubes treated with 0.25% Ethylene Diamine Tetraacetic Acid (BD Vacutainer Systems, Plymouth, UK) and stored at −20°C until DNA isolation that was conducted 2 weeks following collection. DNA was isolated from the whole blood samples using Wizard Genomic DNA Extraction Kit (OMGA-Bio-Tek, Inc., Madison., WI, USA), according to the manufacturer’s instructions, and then stored at –20°C. The quality of the obtained DNAs was tested using agarose gel electrophoresis (Cleaver Scientific Ltd, Belgium). The studied genetic regions were amplified after obtaining the specific primers that target exon 7 of*CSN2* and exon 17 of the *CSN1S1* genes (Table-1) [11,18]. Polymerase chain reaction (PCR) amplifications mixture contained nuclease-free water (10 μL), template genomic DNA (100 ng/μL) (2 μL), two μL from each of the prepared primers, and *Taq* DNA polymerase (4 μL [5 U/μL]). Primer sequences and annealing temperature are shown in Table-1. The PCR reaction mixtures were performed using a thermal cycle parameter at 95°C for 5 min as initial denaturation step followed by 33 cycles at 95°C for 30 s, 40 s annealing and extension each at 72°C, and a final extension step at 72°C for 7 min (Table-1). The PCR products were visualized on a 2% agarose gel stained with bromide (Bio Basic Inc., Canada) and visualized under ultraviolet light.

**Table 1:** The primers information used in this study.

Gene	Primers (5’→3’)	TM (°C)	Polymerase chain reaction product (bp)	Reference
AlphaS1-casein (*CSN1S1*)	F: CACTGTTGCTTTTTCAATGGC R: AAGGCAACAATATGCAGTCATTT	56	223	[11]
Bata-casein (*CSN2*)	F: CTTCTTTCCAGGATGAACTCC R: GACTTACAAGAATAGGGAAGG	52	510	[18]

TM=Annealing temperature

### Sequencing analysis

The primers (Table-1) used for amplification were also included for obtaining the nucleotide sequences. The PCR products of the different genotype patterns of the *CSN1S1* and *CSN2* genes were purified and sequenced by Macrogen Incorporation (Seoul, South Korea) to identify the SNPs found in these different genotype patterns. The nucleotide sequences and alignments were analyzed by BioEdit software version 5.0.6. [19].

### Statistical analysis

The genotype and allelic frequencies of the *CSN2* and *CSN1S1* loci, and their probable deviations from Hardy-Weinberg equilibrium were evaluated by Pop-Gene32 package version 1.31 programs [20]. The least-squares method applied in the mixed model procedure of SAS/STAT® software (SAS Institute Inc., Cary, NC, USA, v9.1) was used to investigate the single and combined impact of *CSN2* and *CSN1S1* genes on the studied traits using two statistical models as described below:

The first model was used to estimate the impact of the detected mutations on milk production traits analysis was:

Yijklmno=μ+*CSN1S1*i+*CSN2*j+Pk+D(S)I+SYm+βnDWn+(*CSN1S1×CSN2*)ij+eijklmno

Where:

- Yijklmno=The value of each studied trait
- μ=Overall mean of the total milk yield (TMY) or test day milk (TDM) yield
- *CSN1S1*i=The effect of the i^th^ genotype at *CSN1S1* locus (i=TT and TC)
- *CSN2*j=The effect of the j^th^ genotype at *CSN2* locus (j=CC and CA)
- Pk=The effect of the k^th^ parity or number of lambing (K=1, 2,3,4,5 and 6)
- D(S)l=The effect of I^th^ dams within sires (l=1, 2-30); (Random effect)
- SYm=Fixed effect of the m^th^ year-season of lambing (m=1-5)
- βn=Regression coefficient dam weight at lambing
- DWn=Dam weight at lambing as a covariate
- (*CSN1S1×CSN2*)ij=Interaction between *CSN1S1* genotypes and *CSN2* genotypes (ij=TTCC, TTCA, TCCC, and TCCA);
- eijklmno=random errors with the assumption of N (0, σ^2^).

The second model used to estimate the effects of the mutations on milk composition traits analysis was:

Yijklmno=μ+*CSN1S1*i+*CSN2*j+Pk+Sl+TOBm +AGEn+βoDWo+βpTDMp+(*CSN1S1×CSN2*) ij+eijklmnopq

Where:

Yijklnmopq=The studied traits
μ=Overall mean of Fat %; protein %, solid not fat (SNF) %, Total solids, lactose %, and density (g/cm^2^)
*CSN1S1*i=Fixed effect of the i^th^ genotype at *CSN1S1* locus (i=TT and TC)
*CSN2*j=Fixed effect of the j^th^ genotype at *CSN2* locus (j=CC and CA)
Pk=Fixed effect of the k^th^ parity or number of lambing (k=1, 2,3,4,5 and 6)
Sl=Random effect of l^th^ sires (l=1, 2…, 31)
TOBm=Fixed effect of the m^th^ type of birth (m=single and twin)
AGEn=Fixed effect of the n^th^ age of dam (n=2-7)
βo=Linear regression coefficient dam weight at lambing
DWo=Dam weight at lambing as a covariate
βp=Linear regression coefficient TDM
TDMp=TDM covariant.
(*CSN1S1×CSN2*)ij=Interaction between *CSN1S1* genotypes and *CSN2* genotypes (ij=TTCC, TTCA, TCCC, and TCCA)
eijklmnopq=Random errors with the assumption of N (0, σ^2^).

All statistical comparisons between genotypes and traits were considered to be significant when p<0.05.

## Results

### Descriptive statistics

In the obtained data that measured on the commercial flocks, the necessary test statistics were generated (Table-2), including; the means and their standard errors, coefficients of variation (CV), which are presented in Table-2. The standard errors of the means of milk production and composition were very low, while the other statistics such as CV indicated the high diversity within the studied flocks indicting high CV for fat percentage and milk production but relatively average coefficient of variation for lactose and protein percentages, that promoting and escalating the selection gain in the two studied traits recorded in these flocks.

**Table 2:** Milk production and composition simple statistics calculations in the studied population.

Traits	No. of records	Mean	SE	CV (%)
Milk production traits (kg)				
TMY	576	105.9	2.09	47.3
TDM	576	0.933	0.02	40.4
Milk composition trait				
Fat%	917	5.80	0.05	25.7
SNF%	986	9.74	0.03	8.30
Protein%	986	3.90	0.02	13.0
Lactose%	986	5.10	0.02	13.9
Density g/cm^2^	986	34.3	0.10	9.4

TMY=Total milk yield, CV=Coefficient of variation (%), TDM=Test-day milk, SNF=Soluble-not-fat, SE=Standard error

### Sequence analysis of the *CSN1S1* and *CSN2* genes

**Figure-1:**
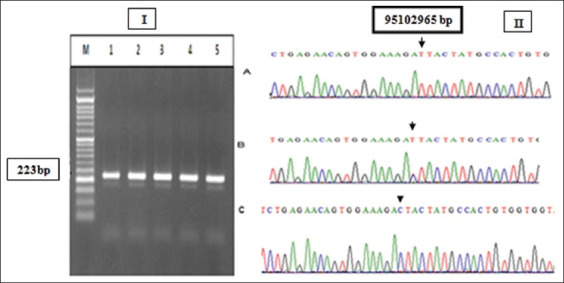
(I) Agarose gel electrophoresis stained with ethidium bromide showing the polymerase chain reaction (PCR) product of as1-Casein gene (*CSN1S1*). M: 50-bp ladder. Lanes 1-5: 223-bp. (II) The sequence of *CSN1S1* PCR product, (A) TT genotype, (B) TC genotype, and (C) CC genotype at position 85102965bp of the gene located at the 6^th^ ovine chromosome.

**Figure-2:**
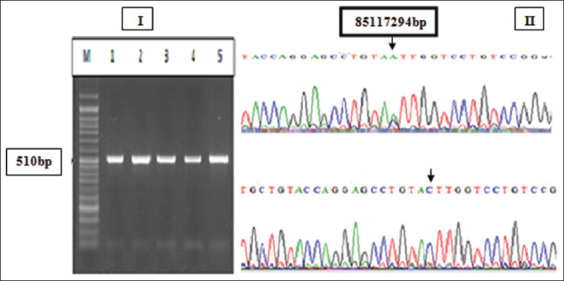
(I) Agarose gel electrophoresis stained with ethidium bromide showing the polymerase chain reaction (PCR) product of beta-casein (*CSN2*) gene. M: 50-bp ladder. Lanes 1-5: 510-bp. (II) The sequence of the PCR product results of the *CSN2* gene a mutation variant located at 8511729bp on the 6^th^ ovine chromosome, (A) AC genotype and (B) The CC genotype.

The regions spanning from exon number 17 to the 3’ flanking region of the *CSN1S1* ovine gene and that spanning from exon number 7 to the 3’ flanking region of the *CSN2* gene were amplified. The PCR amplified product of the *CSN1S1* gene had a size of 223bp, while that of *CSN2* gene was 510bp (Figures-1 and 2). BioEdit software was performed for the alignment process and analysis of the sequences generated from the PCR products (Figures-1 and 2). The sequence analysis of exon 17 in *CSN1S1* gene revealed a missense mutation (C>T) at 14079bp (relative to the gene size) that caused a change in the codons ACT/ATT and new amino acid formation (Threonine/Isoleucine). In exon 7 of the *CSN2* gene, a missense mutation (C>A) at 6083bp caused codon change (CTT/ATT) and new amino acid formation (Leucine/Isoleucine) was appeared (Figures-1 and 2). Three genotypes in *CSN1S1* locus were observed (TT, TC, and CC) while only two Ac and CC were detected in *CSN2* genetic locus.

### Allelic and genotypic frequencies of *CSN1S1* and *CSN2* mutations

The *CSN1S1* and *CSN2* genes and allele frequencies are summarized in Table-3. The frequencies of T and C alleles at the locus studied in *CSN1S1* gene were 0.85 and 0.15, respectively, being the TT genotype as the most common genotype (0.72), followed by TC (0.27). In contrast, CC genotype was rarely found in the Awassi sheep (0.01) genome. For the *CSN2* gene, the frequency of CC genotype was of high prevalence (0.89), while AC was 0.11 and AA was absent in the studied Awassi sheep population. Overall, A allele was of lower frequency than C allele (0.05 and 0.95, respectively). According to Chi-square test results that investigated the equilibrium of Hardy-Weinberg indicated all genotypic frequencies in the studied population to be in equilibrium (p<0.05), suggesting that the *CSN1S1* and *CSN2* gene in the investigated population were not influenced by the selection of other evolutionary forces (Table-3).

**Table 3:** *CSN1S1* and *CSN2* genotypic and allelic frequencies in Awassi sheep.

Gene^1^	Genotype	Observed number	Expected number	Genotype frequency	Allele	Allele frequency	χ^2^
*CSN1S1* (n=158)	TT	114	115.3	0.72	T	0.85	0.74^ns^
	TC	42	39.3	0.27	C	0.15	
	CC	2	3.34	0.01			
*CSN2* (n=160)	CC	143	143.5	0.89	C	0.95	0.50^ns^
	CA	17	16.1	0.11	A	0.05	

1 *CSN1S1*=AlphaS1 casein, *CSN2*=Beta-casein, n=Number of animals, ns=Non-significant (p>0.05)

### The impact of *CSN1S1* and *CSN2* genotypes and some fixed on Awassi milk traits

The environmental factors that may affect the composition and the amount of milk produced are presented (Table-4). A highly significant impact (p<0.05) of parity, year-season of lambing and Dam within sire, on milk composition and production traits were observed. However, TDM and TMY were not affected by the *CSN1S1* gene genotypes (p>0.05), while *CSN2* gene significantly affected TDM and TMY (p<0.05). A significant interaction effect was observed between *CSN1S1* and *CSN2* loci on TDM and TMY (p<0.04 and 0.05).

**Table 4:** P values for the studied milk traits.

Trait factors	Milk Production^1^		Milk Components				
	
	TMY (kg)	TDM (kg)	Fat%	SNF%	Protein%	Lactose%	Density, g/cm^2^
*CSN1S1*	0.945	0.368	0.576	0.040	0.960	0.05	0.115
*CSN2*	0.037	0.001	0.615	0.489	0.474	0.940	0.572
Dam (Sire)	<0.0001	<0.0001					
Year	<0.0001	0.002					
Parity	0.004	0.005	0.569	0.039	0.473	0.063	0.069
Sire			0.004	<0.0001	0.0002	0.009	0.001
TOB			0.055	0.054	0.070	0.560	0.063
Age of dam			0.067	0.042	0.022	0.045	0.069
*CSN1S1*×*CSN2*	0.05	0.042	0.025	0.012	0.309	0.161	0.162
Dam weight at lambing	0.532	0.020	0.031	0.005	0.207	0.045	0.120
Test day milk			0.0003	<0.0001	<0.0001	0.132	<0.0001

1 Mixed-model results of the included fixed effects, TDM=Test-day milk, SNF=Solids-non-fat, TMY=Total milk yield, *CSN1S1*=Alpha S1-casein, *CSN2*=Beta-casein

Furthermore, SNF% and lactose% were significantly (p<0.05) affected by *CSN1S1* mutation locus. The *CSN2* gene variants were non-significantly affected any of milk composition investigated traits (Table-4). Milk composition was affected by sire (p<0.05), and parity was significantly affected SNF percentage (p<0.05). Type of birth has no significant effect on composition milk traits (p>0.05), while age of ewe significantly affected SNF%, protein%, and lactose% (p<0.05). A significant interaction effect was observed on fat% and SNF% at p<0.025 and 0.012, respectively.

### Single and combined effects of *CSN1S1* and *CSN2*

The single and combined impacts of *CSN1S1*and *CSN2* genotypes on milk productivity are shown in Table-5. The single gene effect of *CSN1S1* indicated no association (p>0.05) with milk amount produced from Awassi ewes. For *CSN2* gene, the highest milk produced was obtained from those ewes of CA (Table-5) genotype.

**Table 5:** The single and combined effects of *CSN1S1* and *CSN2* genotypes on test day (TDM) and TMY production in Awassi sheep.

Gene	Genotype	n	Least square means (±S.E)	

			TMY (kg)	TDM (kg)
*CSN1S1*	TT	377	137.1±18.6	1.191±0.11
	TC	118	135.2±16.6	1.030±0.12
*CSN2*	CC	455	107.2±12.0^b^	0.796±0.08^b^
	CA	50	165.2±22.0^a^	1.425±0.14^a^
*CSN1S1*×*CSN2*	TTCC	360	95.0±7.71^c^	0.794±0.05^b^
	TTCA	17	175.6±33.0^a^	1.589±0.21^a^
	TCCC	85	119.5±23.0^bc^	0.798±0.15^b^
	TCCA	33	154.7±33.2^ab^	1.262±0.22^a^

TMY=Total milk yield, TDM=Test-day milk. ^a,b,c^ Mean within the same row with superscripts differ according to the indicated level of significance (p<0.05).

The combined effects of the two genes (*CSN1S1×CSN2*) are presented in Table-5. The outcomes from the combined effect of *CSN1S1×CSN2* pointed out that the individuals of TT×CA genotype produced significantly the highest amount of milk (TDM and TDY) produced compared to the other genotypes.

### Single and combined effects of *CSN1S1* and *CSN2* genotypes on milk composition traits and their interactions

The association of different *CSN1S1* and *CSN2* genotypes and their interactions with milk composition are shown in Table-6. The lactose and SNF percentage in Awassi milk was affected by *CSN1S1* gene where TC genotype individuals produced the highest percentage of lactose% and SNF% compared to the TT genotypes (p<0.05). The *CSN2* gene showed no significant (p>0.05) association with all milk composition traits. Compared to their other respective genotypes; TT×CC and TC×CA genotype (*CSN1S1×CSN2*) showed the highest fat%, while TC×AG genotype recorded the highest SNF% (Table-6).

**Table 6:** Single and combined effect of *CSN1S1* and *CSN2* genotypes on milk composition traits in Awassi sheep.

Gene	Genotype	n	Traits least square means (±S.E)				

			Fat %	SNF %	Protein %	Lactose %	Density, g\cm^2^
*CSN1S1*	TT	685	5.53±0.22	9.53±0.12^b^	3.90±0.07	4.98±0.10^b^	33.4±0.46
	TC	223	5.40±0.19	9.80±0.10^a^	3.90±0.06	5.20±0.09^a^	34.2±0.40
*CSN2*	CC	814	5.40±0.16	9.62±0.08	3.75±0.05	5.09±0.07	33.9±0.32
	CA	94	5.53±0.25	9.71±0.13	3.82±0.08	5.08±0.12	33.6±0.53
*CSN1S1*×*CSN2*	TTCC	654	5.75±0.15^a^	9.65±0.07^b^	3.79±0.05	5.06±0.07	33.9±0.29
	TTCA	31	5.32±0.40^ab^	9.41±0.21^b^	3.77±0.13	4.89±0.19	32.8±0.85
	TCCC	160	5.05±0.21^b^	9.59±0.11^b^	3.71±0.07	5.12±0.10	34.0±0.43
	TCCA	63	5.74±0.27^a^	10.01±0.14^a^	3.86±0.09	5.28±0.13	34.4±0.57

## Discussion

This study reported single and combined effect of CSN1S1 and CSN2 genotypes and their possible association with milk composition and Awassi sheep production. Regardless of the essential function of casein genes variants on the economic milk traits, there is a shortage in studies concerning the ovine milk genetic polymorphisms compared with other published work in cattle and goat. Different genotypes and allelic frequencies were observed in *CSN1S1* and *CSN2* genes of the Jordanian commercial Awassi sheep (Table-3).

The T allele was observed of high frequency (0.85) compared to the C allele (0.15) of *CSN1S1* locus in Awassi sheep. Similarly, it was found to be at 0.71 in Switzerland Lacaune sheep [12], 0.85 in Barki, 0.68 Rahmani, and 0.90 Ossimi sheep [21], 0.53 in German East Friesian Dairy [22], 0.65 in Gentile di Puglia and Massese, 0.73 in Comisana, 0.81 in Sopravissana, and 0.89 in Sarda breed [16]. However, the polymorphisms detected in milk traits of Awassi were not associated with *CSN1S1* gene (Table-5). Our results were comparable with the findings published by Giambra *et al*. [12,22] in East Friesian and Lacaune sheep, as they reported milk yield to be not affected by *CSN1S1* genotypes. Milk production of the Spanish Assaf was also not affected by *CSN1S1* genotypes [23]. Protein, fat content, and milk density were non significantly affected by *CSN1S1* variants, whereas lactose and SNF content were affected positively by only the TC genotype (Table-6). However, such evidence has not been consistently found in sheep. Recently Giambra *et al*. [12] showed that *CSN1S1* T allele had a positive significant effect on protein content, while there were no significant differences in fat content, when studying East Friesian and Lacaune sheep breeds.

On the other hand, Calvo *et al*. [23] claimed no association when they tested the influence of *CSN1S1* gene on milk protein, lactose, and fat contents in Spanish Assaf sheep. The strength of the statistical procedures and the problem of false discovery rates and the genotype by environmental interaction, including different fixed or environmental included in the model, may explain the discrepancies about the effect of *CSN1S1* on milk traits reported in our and other studies. Overall the observed effect of *CSN1S1* on some milk composition traits, *CSN1S1* gene variants should be included in selection criteria as a mark­er-assisted selection procedure for improving milk composition.

In the *CSN2* gene, we found C allelic frequency higher than the A allele in Awassi sheep. However, animals with the AA genotype were not observed and the CC genotype prevailed over the CA genotype (Table-2). To the best of our knowledge, the present study associates, for the 1^st^ time, the *CSN2* gene to milk production and composition. The AC genotype was associated with the highest milk production (Table-5), whereas the *CSN2* genotypes had no distinct effect on milk composition traits (Table-6). Considering the effect of *CSN2* polymorphism on the milk yield and quality, Corral *et al*. [14] found the GG genotype to cohort with high milk production. In contrast, the AA genotype was related to an increase in fat and protein percentages in Merino sheep breed.

On the other hand, Giambra *et al*. [12]found the genotypes of the *CSN2* to have no significant effect on milk production and composition in East Friesian and Lacaune sheep. Genotypes combinations reflect the multiple genes effects in a certain quantitative variable [24] which is the interesting portion of our study. A significant interaction impact of the *CSN1S1×CSN2* genotypes on milk production (Table-5) was found. Milk production traits were the highest in Awassi ewes of TT×CA (*CSN1S1×CSN2*) combined genotypes compared to the other genotype. However, our study indicated a significant interaction impact of *CSN1S1*×*CSN2* genotypes on milk composition traits. The statistical results show that ewes of TT×CC and TC×CA genotypes combination had the highest Fat%, and TC×CA genotype combination had the highest SNF% compared to other genotypes (Table-6).

## Conclusion

The remarkable finding of this study was the significant genes combination impact on the Awassi sheep breed milk. The findings illustrated in this study figured out the single gene effect of *CSN1S1* gene polymorphisms to be not associated with milk production traits; however, their interactions with *CSN2* polymorphisms were observed to affect milk production and composition of Awassi sheep. This finding indicates the necessity of including those genes interaction effects as a marker to assist selection strategy for improving Awassi sheep milk productivity. On the other hand, *CSN2* gene polymorphism was associated with high milk production while not associated with milk composition traits. The selection program that targeted the improvement of Awassi milk production in the commercial flocks should include those genes and their interaction as a remarkable way for rapid improvement of milk production and may reduce the generation intervals and genetics gained as a result from the selection. The commercial flocks herd book and DNA banks should be established to facilitate the selection procedure for gaining an accurate selection decision in Awassi sheep flocks.

## Authors’ Contributions

KIJ, AHA: Conceptualization and methodology, validation, formal analysis, investigation, resources, data collection, sequence analysis, drafted, and revised the manuscript. KIJ and AHA: DNA extraction, genotyping, and data analysis. All authors read and approved the final manuscript.
